# Diagnosis of severe scrub typhus infection by next-generation sequencing:a case report

**DOI:** 10.1186/s12879-020-04991-y

**Published:** 2020-04-07

**Authors:** Jie Chen, Xu-dong Zheng, Qi-he Dai, Wei-li Hong, You-peng Li, Rui Chen, Bing-bing Ye, Xiao-jie Mo, Peng Cui, Zhan-wei Ruan

**Affiliations:** 1 Department of Emergency, Third Affiliated Hospital, Wenzhou Medical University, 108 Wansong Road, Zhejiang, 325200 China; 2 BGI-Shanghai, Kangxin Rd, Pudong District, Shanghai, 201321 Guangdong China

**Keywords:** Scrub typhus, Next generation sequencing, Orientia tsutsugamushi, Septic shock, Multiple organ dysfunction

## Abstract

**Background:**

Scrub typhus is an acute febrile illness, which was caused by Orientia tsutsugamushi and transmitted through the bite of chiggers. The diagnosis of scrub typhus could be missed diagnosis due to the absence of the pathognomonic eschar.

**Case presentation:**

A 76-year-old man was hospitalized with fever and kidney injury and was diagnosed of hemorrhagic fever with renal syndrome first. However, the situation of the illness deteriorated into refractory septic shock and multiple organ dysfunction rapidly,although the treatment of anti-sepsis was used in 3rd-5th day. Orientia tsutsugamushi was determined to be the causative pathogen by Next-generation sequencing of his plasma sample in 6th day. Then, the patient was treated with doxycycline and azithromycin and recovered quickly.

**Conclusions:**

Next-generation sequencing was a new diagnostic technology and could identify scrub typhus in accurately and fast without the pathognomonic eschar.

## Background

Scrub typhus is an acute febrile illness caused by Orientia tsutsugamushi (a gram-negative coccobacillus) and transmitted through the bite of chiggers [1]. Themortality of scrub typhus is reported to be as high as 24% in severe cases with multiple organ dysfunction (MODS) [2]. The diagnosis of scrub typhus is difficult due to the absence of the pathognomonic eschar, which is the characteristic clinical manifestations and varies widely (3–93%) [1]. Serology, biopsy, culture, and polymerase chain(PCR) reaction were routine diagnostic methods and had many defects in the diagnosis [3]. For example, the indirect fluorescent antibody test needs a four-fold rise in titers over a 14-day period [4]. Polymerase chain reaction testing is only used as confirmatory test, but not as a screening test, because of multiple pathogenic bacterium in the clinic [5]. The culture of Orientia tsutsugamushi was very difficult and dangerous, and should be accomplished at a special research institution [6]. Lymphohistocytic vasculitis was the pathological hallmark of scrub typhus on skin biopsy, but not definitive [1]. Next-generation sequencing (NGS) technologies have been used in the diagnosis of other pathogens such as *Leptospira santarosai* [7], *Mycobacterium tuberculosis* [8], *Human Immunodeficiency Virus* [9] etc. However, the use of NGS has not been reported in the case of Scrub Typhus. Here, Orientia tsutsugamushi was determined to be the causative pathogen by NGS technologies in a case of MODS, and the results contributed directly to the patient’s dramatic diagnosis and treatment, resulting in a favourable outcome.

## Case presentations

**Fig. 1:**
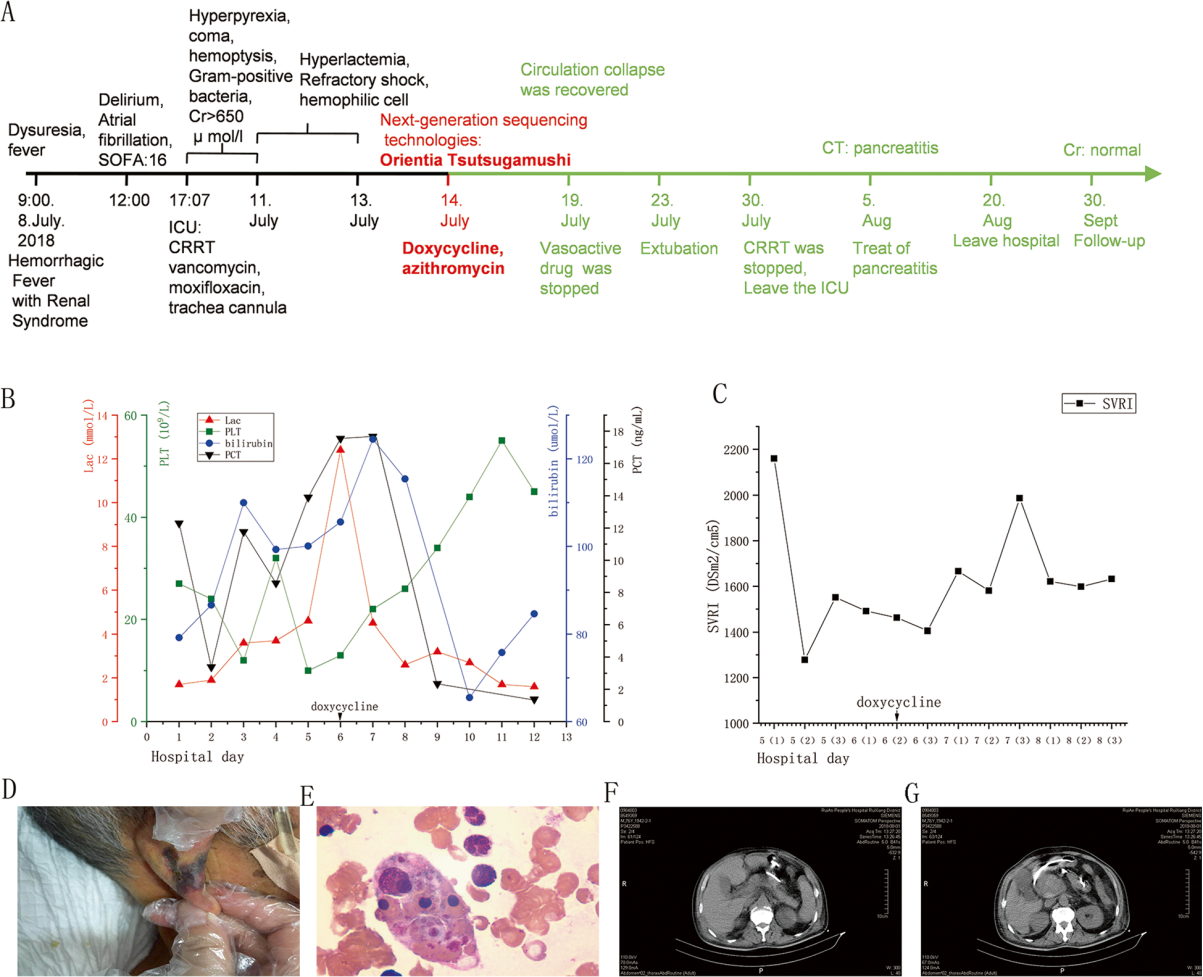
Clinical course of the 76-Year-Old Patient with Scrub Typhus. **a** shows a timeline beginning with the occurrence of the disease in 8.july.2018 and ending after his recovery in 30.sept.2018. Major events during the course of the patient’s illness are described in the line by different colours. Black means deteriorate of the disease, green means recovery of it, red means the turning point. **b** shows clinical laboratory examination values during the patient’s hospitalization. Blood lactic acid curve (Lac, red line), bilirubin (blue line) and procalcitonin (PCT, black line) were decreased with the treatment of doxycycline (black arrows) in 6th day, 7th day and 7th day respectively. Although platelet have been infused by iv in in 4th day, the blood platelet (PLT, green line) was still declined in 5th day. However, the blood platelet was increased obviously in 6th day own to the treatment of doxycycline and azithromycin in 5th day. **c** shows systemic vascular resistance index (SVRI) was increased with the treatment of doxycycline (black arrows) in 6th day, whereas it was reduced in the first 5 days. **d** shows the skin anabrosis on the right ear. Panel E shows the bone marrow cells are hyperplastic obviously, poisoning alteration was existed in the granulocytic cell. The megakaryocytes were obviously proliferated with maturating disorderly. Hemophagocytic phenomenon was pointed by the arrow. Panel F shows blurred shadows around the pancreas, indicated the pancreatic leakage. Besides that, the pancreatic head forms a cystic space with dense shadows, indicated the pancreatic head hematoma by CT

A 76-year-old man, rural mountain inhabitant who frequently encountered mice, had a history of benign prostatic hyperplasia (BPH), was admitted to the Department of Nephrology due to the difficulty in urination and fever for 10 days on 8th July 2018 (Fig. 1a). His body temperature was 38.5 °C, accompanied by fatigue, anorexia, chest tightness, coughing with a small amount of haemoptysis. The Vitals read as, BP: 125/68 mmHg, RR: 20 bpm, HR: 97 Bpm, while slight conjunctival hyperaemia, mild scleral yellow stain, wet voice and wheezing of the lungs, right ear anabrosis (Fig. 1d), no obvious abnormalities in the abdomen and other systems, no bites and eschars were found. Blood test showed white blood cell(WBC) count:8.2 × 10^9^/L, neutrophil: 84.7%, atypical lymphocytes: 1%, platelet: 27 × 10^9^/L, hypersensitive C-reactive protein (CRP):153.65 mg/L, procalcitonin (PCT): 12.3 ng/ml, serum creatinine (CR): 847umol/ L, alanine aminotransferase (ALT): 93 U / L, bilirubin:79.3umol / L. The urine volume was 50 ml/h, urine routine urine protein +, red blood cells +++/HP. CT scan showed a little exudation in the lungs, BPH and no other abnormalities. This patient was diagnosed as “haemorrhagic fever with renal syndrome” caused by Hantavirus first (Fig. 1a).

Unfortunately, the patient developed rapid atrial fibrillation and delirium, and was transferred to ICU, because sequential organ failure assessment score increased from 12 to 16. Continuous renal replacement therapy (CRRT) was used due to high serum creatinine (> 650 μmol/L). In the next 72 h, the patient developed a high fever (39.0 °C), and went into coma. A large amount of haemoptysis and growth of Gram-positive bacteria by blood culture smears were found. We intubated him to protect the airway. However, in the 3rd-5th day after hospitalization (11th July to 13th July), the condition worsened and the patient went into refractory shock (mean arterial pressure: 65 mmHg) although norepinephrine (2.26mcg/kg.min) and adrenaline (0.17mcg/kg.min) were used, low blood vessels tension (minimum of systemic vascular resistance index: 1278 DSm^2^/cm^5^), hyperlactemia (maximum: 12.4 mmol/L), further reduction of platelets (minimum: 10000/ml), hyper-procalcitonin (maximum: 17.68 ng/mL), hyperbilirubinemia (maximum: 124.5 μmol/L) (Fig. 1b, c), Epidemic haemorrhagic fever antibody was negative, hemophagocytic phenomenon in the bone marrow (Fig. 1e) in spite of organ support therapy, yet anti-sepsis treatment was given. (Fig. 1a).

**Fig. 2:**
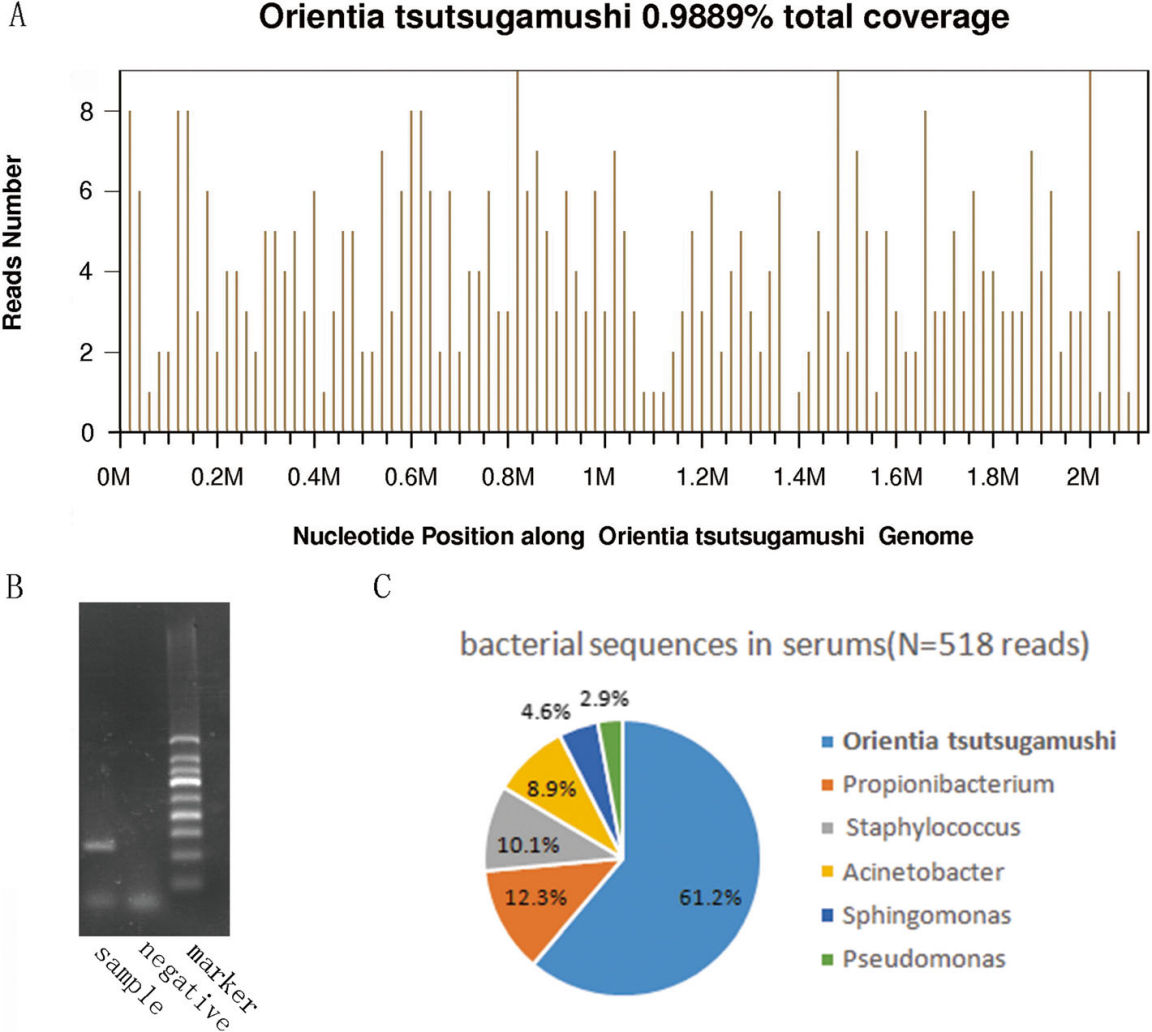
Confirmation of *O. Tsutsugamushi* specific amplification from plasma by next-generation sequencing. **a** shows the reads mapped to *O. Tsutsugamushi* derived from NGS data. A total of 317 reads mapped to *O. Tsutsugamushi* in the reference database which contains about 8000 pathogen genomes, and got a total coverage of 0.99% respectively. **b** shows the confirmation of *O. Tsutsugamushi* specific amplification by PCR. The primer was 5′-AACTGATTTTATTCAACTAATGCTGCT-3′ and 5′-TATGCCTGAGTAAGATACRTGAATRGAATT-3′. The 118 bp PCR products was detected in case sample. Lane 1: sample case, Lane 2: negative control, Lane 3: The DNA ladder was DL2000 from TAKARA. **c** shows the the distribution of bacterial sequences (*N* = 518 reads) identified in the patient’s plasma included Orientia tsutsugamushi(*N* = 317;61.20%), propionibacterium, staphylococcus, acinetobacter, sphingomonas, pseudomonas

Fortunately, on the 6th day after hospitalization (14th July), Orientia tsutsugamushi was determined to be the causative pathogen by NGS of the patient’s plasma sample. High-quality sequencing data were generated by removing low-quality, and short (length < 35 bp) reads, followed by computational subtraction of human host sequences mapped to the human reference genome (hg19) using Burrows-Wheeler Alignment. The remaining data by removal of low-complexity reads were classified by simultaneously aligning to four Microbial Genome Databases, consisting of virus, bacteria, fungi, and parasites. A total of 317 sequences of Orientia tsutsugamushi were detected in plasma sample with a total coverage of 0.99% (Fig. 2a). Orientia tsutsugamushi specific amplification was detected from plasma sample by PCR (Fig. 2b). The distribution of bacterial sequences (*N* = 518 reads) was identified in the patient’s plasma including Orientia tsutsugamushi (*N* = 317, 61.20%), Propionibacterium, staphylococcus, Acinetobacter, sphingomonas, pseudomonas (Fig. 2c). Then, the anti-infection regime was changed to doxycycline (0.1 g oral bid) and azithromycin (0.5 g intravenous drops qd). After 5 days of treatment (19th July), the circulation collapse was recovered and the vasoactive drug was stopped. After 9 days of the treatment (23th July), respiratory failure was corrected and tracheal intubation was removed. The patient was removed haemodialysis and returned to the nephrology ward successfully on 30th July. Then, the patient had abdominal distension, elevated blood amylase, pancreatic exudation and haemorrhage by CT in 5th Aug (Fig. 1f, g) and anti-pancreatitis treatment was timely. After the patient recovered, he was then discharged on 20th Aug and returned to normal serum creatinine in 30th Sep by follow-up (Fig. 1a).

## Discussion and conclusion

Scrub typhus is a natural epidemic caused by Orientia tsutsugamushi, spread globally, mainly in the Asia-Pacific region [1, 3, 5] and is considered as the world’s leading rickettsial infection, threatening the health of one billion people every year and causing more than one million deaths [10]. The hosts of Orientia tsutsugamushi are larvae and rats, while it could also infect humans and spread through the sputum in grass or soil on the floor [2]. Indeed, this patient lived on the mountain side where mice often appeared in Eastern part of china.

Scrub typhus is characterised mainly by ascended vascular permeability directly co-related with the bacteria count, damaged endothelial cell junctions in the small and medium blood vessels due to the escalated TNF-α [3]. The typical clinical manifestation of scrub typhus are the fever and eschar. Severe Scrub typhus infection could affect a variety of systems and lead to multiple complications, including meningitis, acute lung injury, myocarditis, hepatitis, acute renal failure, pancreatitis, disseminated intravascular coagulation, septic shock, and MODS [2, 8]. Unfortunately, because this patient had no specific eschar, he was misdiagnosed as “haemorrhagic fever with renal syndrome” previously. Moreover, condition still deteriorated into refractory septic shock and MODS rapidly, although CRRT, tracheal intubation and antibiotics were used during 3rd -5th days.

Scrub typhus might be easily misdiagnosed as other febrile diseases due to non-specific symptoms except the hidden eschar. Laboratory diagnosis is mainly done with serology, molecular assays, pathogen characterization and tissue culture,which is only supported by large scale laboratories. Serology would be positive after 7–10 days of illness and has poor sensitivity and specificity due to the lack of species identification techniques in the market. Besides that, cross-reactions among closely related members should be noticed [4]. Molecular assays (such as PCR tests) have the advantages of rapid and sensitive diagnosis when an eschar is still present. However, it is difficult to apply on time when the eschar is negative and the initial diagnosis is missed. Tissue biopsy, culture, and pathogen characterization require special laboratories to avoid biosafety risks [3].

NGS technologies have been used in the diagnosis of other pathogenesis widely, which contained 4061 whole genome sequence of viral taxa, 2473bacteral genomes or scaffolds, 199 fungi related to human infection, and 135 parasites associated with human diseases [7–9]. However, the use of NGS has not been reported in the case of Scrub Typhus. Indeed, in the 6th day after hospitalization (14th July), Orientia tsutsugamushi was determined to be the causative pathogen by NGS. Scrub typhus could also be treated effectively if it could be diagnosed in time [1, 11]. Doxycycline has been proved to the preferred drug in the treatment of scrub typhus, and intravenous doxycycline isolation or enteral doxycycline combined with intravenous azithromycin is the better choice in severe cases with shock or intestinal absorption difficulties [2]. Our patient got the combination therapy and recovered quickly.

In summary, it was difficult to diagnose of Scrub typhus timely owing to the lack of specific eschar and many limitations in conventional diagnostic methods. NGS was a new diagnostic technology which could identify Scrub typhus in accurately and fast and wouldbe a promising critical tool to find the aetiology of multiple organs failure or septic shock.

## Supplementary information

**Additional file 1.**

## Data Availability

All data generated or analyzed during this study are included in this published article.

## Abbreviations

BPH: Benign prostatic hyperplasia
DNA: Deoxyribonucleic acid
ICU: Intensive care unit
Lac: Lactic acid
MODS: Multiple organ dysfunction
NGS: Next generation sequencing
PCR: Polymerase chainreaction
PCT: Procalcitonin
PLT: Platelet
SOFA: Sequential organ failure assessment

## References

[1] Weitzel T, Dittrich S, Lopez J, Phuklia W, Martinez-Valdebenito C, Velasquez K (2016). Endemic scrub typhus in South America. N Engl J Med.

[2] Peter JV, Sudarsan TI, Prakash JA, Varghese GM (2015). Severe scrub typhus infection: Clinical features, diagnostic challenges and management. World J Crit Care Med.

[3] Abdad MY, Abou Abdallah R, Fournier PE, Stenos J, Vasoo S. A Concise Review of the Epidemiology and Diagnostics of Rickettsioses: Rickettsia and Orientia spp. J Clin Microbiol. 2018;56(8):e01728–17. 10.1128/JCM.01728-17 PMC6062794 29769278

[4] Lim C, Blacksell SD, Laongnualpanich A, Kantipong P, Day NP, Paris DH (2015). Optimal cutoff titers for indirect immunofluorescence assay for diagnosis of scrub typhus. J Clin Microbiol.

[5] Le Viet N, Laroche M, Thi Pham HL, Viet NL, Mediannikov O, Raoult D (2017). Use of eschar swabbing for the molecular diagnosis and genotyping of Orientia tsutsugamushi causing scrub typhus in Quang Nam province, Vietnam. PLoS Negl Trop Dis.

[6] Blacksell SD, Robinson MT, Newton PN, Day NPJ (2019). Laboratory-acquired scrub typhus and murine typhus infections: the argument for a risk-based approach to biosafety requirements for Orientia tsutsugamushi and rickettsia typhi laboratory activities. Clin Infect Dis.

[7] Wilson MR, Naccache SN, Samayoa E, Biagtan M, Bashir H, Yu G (2014). Actionable diagnosis of neuroleptospirosis by next-generation sequencing. N Engl J Med.

[8] Hassibi A, Manickam A, Singh R, Bolouki S, Sinha R, Jirage KB (2018). Multiplexed identification, quantification and genotyping of infectious agents using a semiconductor biochip. Nat Biotechnol.

[9] Inzaule SC, Ondoa P, Peter T, Mugyenyi PN, Stevens WS, de Wit TFR (2016). Affordable HIV drug-resistance testing for monitoring of antiretroviral therapy in sub-Saharan Africa. Lancet Infect Dis.

[10] Weitzel T, Aylwin M, Martinez-Valdebenito C, Jiang J, Munita JM, Thompson (2018). Imported scrub typhus: first case in South America and review of the literature. Trop Dis Travel Med Vaccines.

[11] Kim SJ, Chung IK, Chung IS, Song DH, Park SH, Kim HS (2000). The clinical significance of upper gastrointestinal endoscopy in gastrointestinal vasculitis related to scrub typhus. Endoscopy..

